# The Pyroptosis-Related Signature Predicts Prognosis and Indicates Immune Microenvironment Infiltration in Gastric Cancer

**DOI:** 10.3389/fcell.2021.676485

**Published:** 2021-06-11

**Authors:** Wei Shao, Zongcheng Yang, Yue Fu, Lixin Zheng, Fen Liu, Li Chai, Jihui Jia

**Affiliations:** ^1^Key Laboratory for Experimental Teratology of The Chinese Ministry of Education, Department of Microbiology, School of Basic Medical Science, Cheeloo College of Medicine, Shandong University, Jinan, China; ^2^Key Laboratory of Infection and Immunity of Shandong Province, School of Basic Medical Science, Cheeloo College of Medicine, Shandong University, Jinan, China; ^3^School of Stomatology, Cheeloo College of Medicine, Shandong University, Jinan, China; ^4^School of Medicine, Cheeloo College of Medicine, Shandong University, Jinan, China; ^5^Shandong University-Karolinska Institutet Collaborative Laboratory for Cancer Research, Jinan, China

**Keywords:** pyroptosis, gastric cancer, prognosis, tumor microenvironment, immunotherapy, lasso-cox regression

## Abstract

Gastric cancer (GC) is one of the leading causes of cancer-related deaths and shows high levels of heterogeneity. The development of a specific prognostic model is important if we are to improve treatment strategies. Pyroptosis can arise in response to *H. pylori*, a primary carcinogen, and also in response to chemotherapy drugs. However, the prognostic evaluation of GC to pyroptosis is insufficient. Consensus clustering by pyroptosis-related regulators was used to classify 618 patients with GC from four GEO cohorts. Following Cox regression with differentially expressed genes, our prognosis model (PS-score) was built by LASSO-Cox analysis. The TCGA-STAD cohort was used as the validation set. ESTIMATE, CIBERSORTx, and EPIC were used to investigate the tumor microenvironment (TME). Immunotherapy cohorts by blocking *PD1*/*PD-L1* were used to investigate the treatment response. The subtyping of GC based on pyroptosis-related regulators was able to classify patients according to different clinical traits and TME. The difference between the two subtypes identified in this study was used to develop a prognosis model which we named “PS-score.” The PS-score could predict the prognosis of patients with GC and his/her overall survival time. A low PS-score implies greater inflammatory cell infiltration and better response of immunotherapy by *PD1*/*PD-L1* blockers. Our findings provide a foundation for future research targeting pyroptosis and its immune microenvironment to improve prognosis and responses to immunotherapy.

## Introduction

Gastric cancer (GC) is the world’s third-highest cause of death by cancer (Smyth et al., 2020). Each year, at least 1 million people worldwide are diagnosed with GC (Thrift and El-Serag, 2020). This disease is mostly detected in its advanced stages and abnormalities in the tumor microenvironment (TME) may lead to widespread tumor heterogeneity. Furthermore, there is significant heterogeneity with regards to the response of GC patients to therapy. So, the prognosis has not been improved (Bray et al., 2018). Pyroptosis refers to the cleavage of gasdermins via classical and non-classical pathways and can lead to the continuous expansion of cells until the cell membrane ruptures and causes the release of the cell contents, thus triggering a strong inflammatory response (Shi et al., 2015; Ding et al., 2016; Rogers et al., 2017; Xia et al., 2019; Broz et al., 2020; Zhang Z. et al., 2020; Zhou et al., 2020). Pyroptosis plays an important role in antagonizing infection and endogenous danger signals. Pathogens such as *H. pylori* or chemotherapy drugs can cause pyroptosis in patients with GC. Pyroptosis creates a tumor-suppressive environment by releasing inflammatory factors, however, it can also weaken the body’s immune effect on tumor cells and accelerate tumor growth in different cancers (Zaki et al., 2010; Ellis et al., 2011; Chen et al., 2012; Ma et al., 2016). However, the effect of pyroptosis on the prognosis of GC is not clear.

The classification of GC patients by next-generation sequencing is a novel method that can quickly identify cancer characteristics and inform us about the most appropriate treatment strategies. Drug treatment already uses *HER2* as a predictive biomarker (Smyth et al., 2020). But the value of HER2 in the prognosis of GC remains controversial (Tanner et al., 2005; Park et al., 2006; Gravalos and Jimeno, 2008; Ruschoff et al., 2010; Begnami et al., 2011; Yoon et al., 2012). *PD-L1*, as an immunotherapy index, also requires further verification (Shitara et al., 2018). Other biomarkers are currently being evaluated. Due to the lack of subgroup classifications, clinical practice cannot be guided by molecular subtypes. Therefore, there is an urgent need for the development of an effective gene signature to indicate prognosis and to guide clinical treatment, especially with regards to targeted therapy and immunotherapy.

In the present study, we aimed to build a scoring model (that produced the PS-score) by classifying GC patients based on pyroptosis-related regulators to predict prognosis and guide clinical treatment. We clustered 618 patients with GC according to pyroptosis-related genes and identified two types of pyroptosis-related subtypes that were related to prognosis and immune infiltration. On this basis, the PS-score can be determined by constructing a pyroptosis-related model using the LASSO-Cox method. This score is able to predict prognosis, immune infiltration, and immunotherapy response. Our findings indicate the potential connection between pyroptosis, prognosis, the immune microenvironment, and the response to immunotherapy of GC patients.

## Materials and Methods

### Sources of Gastric Cancer Datasets and Preprocessing

The workflow chart (Supplementary Figure 1) describes which samples were utilized at each stage of statistical analysis. Microarray data from Affymetrix^®^ was obtained from Gene Expression Omnibus (GEO). Batch effects from non-biological technical biases were corrected by using the “ComBat” algorithm of the “SVA” package. All of the clinical information used in this study are publicly available in the GEO database. As to datasets in TCGA, RNA sequencing data (FPKM value) of gene expression were downloaded from UCSC. Patients without survival information were removed from further analysis.

The waterfall function within the “maftools” package was applied to present the mutation landscape. Patients in the immunotherapy datasets were from GSE78220, NCT01358721, and IMvigor210 treated with Pembrolizumab, Nivolumab, and Atezolizumab, respectively (Choueiri et al., 2016; Hugo et al., 2016; Mariathasan et al., 2018).

### Human Clinical Specimens

Twenty-two pairs of RNA samples of GC and adjacent non-tumor tissues were obtained from Jinan Central Hospital, Shandong, P. R. China. The study protocol was approved by Shandong University Research Ethics Committee.

### Defining Pyroptosis-Related Regulators

In previous research, Shi et al. (2015) found that *Caspase 1* (*CASP1*) and *Caspase 4/5* (*CASP4/5*) could specifically cleave *Gasdermin D* (*GSDMD*) and that the cleaved form of *GSDMD* is necessary for pyroptosis; these findings were subsequently confirmed by He et al. (2015). Later, Orning et al. (2018) found that increasing the concentration of *Caspase 8* (*CASP8*) was another effective way cause the cleavage of *GSDMD*. Since then, a large number of studies have begun to explore the role of gasdermins in cells. Research has found that *Caspase 3* (*CASP3*) and *Granzyme B* (*GZMB*) are capable of cleaving *Gasdermin E* (*GSDME*), thus converting cell apoptosis into pyroptosis (Rogers et al., 2017; Zhang Z. et al., 2020). Apoptosis can also be converted into pyroptosis when *Gasdermin B* (*GSDMB*) is cleaved by *Granzyme A* (*GZMA*) (Zhou et al., 2020). We therefore chose the 11 genes (*CASP1*, *CASP3*, *CASP4*, *CASP5*, *CASP8*, *GSDMB*, *GSMDC*, *GSDMD*, *GSDME*, *GZMA*, *GZMB*) that related closely to cell pyroptosis as pyroptosis-related regulators.

### Consensus Clustering

Consensus clustering was applied to identify distinct pyroptosis-related patterns relating to the expression of pyroptosis regulators by the k-means method. The number of clusters, and their stability, were determined by the consensus clustering algorithm using the “ConsensuClusterPlus” package (John Hartigan, 1979). We performed 1,000 times repetitions to guarantee the stability of our classification (Wilkerson and Hayes, 2010).

### Gene Set Variation Analysis (GSVA)

We performed GSVA enrichment analysis in heatmap by the “GSVA” R packages (Hanzelmann et al., 2013). We downloaded “c2.cp.kegg.v6.2.symbols” from the MSigDB database to carry out GSVA analysis. An adjusted *P* < 0⋅05 was considered to indicate statistical significance between different subgroups by the “limma” package.

### Differentially Expressed Genes (DEGs)

We used the empirical Bayesian approach of the “limma” package to obtain DEGs (Ritchie et al., 2015). The significance criteria for selecting DEGs was set as an adjusted *P* < 0⋅05 and an absolute value of Log2 FC ≥ 0⋅8.

### TME Cell Infiltration

We used the CIBERSORTx algorithm and EPIC to quantify the proportions of immune cells. For CIBERSORTx, we uploaded the normalized gene expression data to the web portal using LM22 signature and 1,000 permutations (Newman et al., 2015). EPIC is a web-based analytical and discovery platform for analyzing mass cytometry data from immune cells in a standardized manner (Yeo et al., 2020). Tumor purity scores were estimated by the “ESTIMATE” package (Yoshihara et al., 2013; Yang et al., 2019).

### Multivariate Cox Regression Analyses

We used univariate Cox regression and multivariate Cox regression analyses for overall survival (OS) in four GEO datasets described earlier. A false discovery rate (FDR) < 0.05 was used as a statistical boundary. The results of multivariate prognostic analysis for pyroptosis-related subgroups were acquired by application of the “forestplot” package.

### The Establishment of a PS-Score Scoring Model and Prognostic Analysis

We established an efficient prediction model using LASSO−Cox analysis. OS was then used to derive the most useful predictive features from the training cohort (Lossos et al., 2004). PS-score =

$$\sum_{i=1}^{k}\beta iPi$$

where k, βi, Pi represented the number of signature genes, the coefficient index, and the gene expression level, respectively. The cut-off point was determined using the “survminer” package. We used Kaplan-Meier survival curves to identify the ability of the model to distinguish different subtypes of patients and time-dependent receiver operating characteristic curves (ROC curves) to determine the efficiency of the model. The C-index was calculated by the “survcomp” package and compared using the “cindex.comp” package.

### RNA Extraction and Real-Time PCR

Total RNA was extracted by Trizol reagent (Invitrogen, Carlsbad, CA, United States) according to the manufacturer’s protocol. Subsequently, the extracted RNA was reversetranscribed using PrimeScript RT reagent Kit with gDNA Eraser (Takara, Japan). The cDNAs were subjected to SYBR Green-based real-time PCR analysis. The primers used in real-time PCR assays were listed in Supplementary Table 1.

### Statistical Analysis and Cut-Off Value

Correlation coefficients were computed by Spearman’s and distance correlation analyses. Log-rank tests were utilized to identify the significance of differences in survival curves. The cut-off value mentioned in this article was 1⋅258 as the best cut-off value from the “survminer” package. ROC curves, time-dependent ROC curves, and the area under curves (AUC) were derived using the “pROC” and the “timeROC” packages, respectively. Comparisons of the integrated area under the curves (IAUC) were carried out with the “iauc.comp” package. The “RCircos” package allowed us to plot the copy number variation landscape of pyroptosis regulators in 23 pairs of chromosomes (Mayakonda et al., 2018). Analyses between the two groups were performed using the Wilcox test. The Kruskal Wallis test was also used to compare three or more groups. Gene expression data, and all statistical analyses were carried out in R 4.0.0, GraphPad Prism 8, and SPSS26 software. Clinical features were compared by Chi-square tests or Fisher’s exact tests where appropriate. All statistical *P* values are two-side and *P* < 0⋅05 represents statistical significance.

## Results

### Overview of Genetic Changes and Expression Variations of Pyroptosis-Related Regulators in GC

**FIGURE 1:**
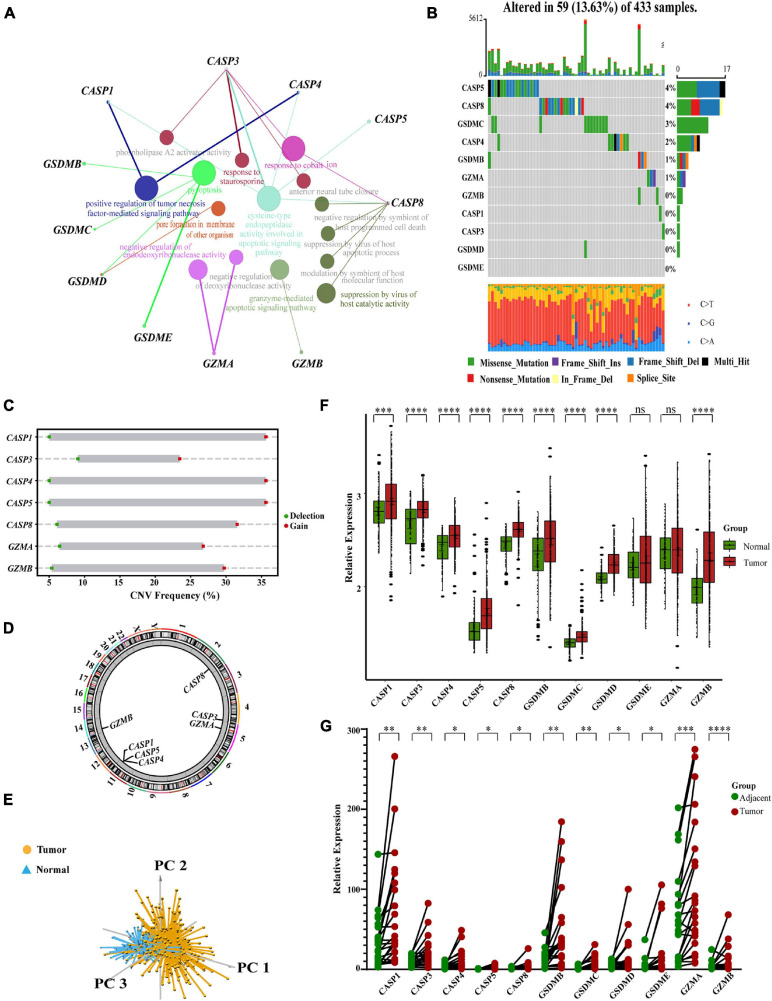
Characteristics and differences of pyroptosis-related regulators in GC. **(A)** An aggregate of the potential biological interaction of pyroptosis-related regulators from STRING platform. **(B)** The landscape of mutation profiles in 433 gastric cancer patients from TCGA-STAD cohort. Every waterfall plot represented mutation information of each pyroptosis-related regulator. Corresponding colors had annotations at the bottom which mean different mutation types. The above barplot showed mutation burden. The right numbers represented mutation frequency individually. **(C)** CNV frequency of pyroptosis-related regulators in GSE62717 cohort. The height of the columns showed proportions of different types. CNV, copy number variations. **(D)** The location of CNV alteration of pyroptosis-related regulators on chromosomes by GSE62717 cohort. CNV, copy number variations. **(E)** Principal component analysis for the expression of pyroptosis-related regulators to distinguish tumors (*n* = 300) from normal samples (*n* = 100) in GSE66229 cohort. **(F)** The expressions of pyroptosis-related regulators between normal tissues (*n* = 100) and gastric tissues (*n* = 300) in GSE66229 cohort (Wilcox test, ^∗^*P* < 0.05; ^∗∗^*P* < 0.01; ^∗∗∗^*P* < 0.001; ^****^P < 0.0001; ns, not statistically significant). **(G)** The mRNA levels of pyroptosis-related regulators in twenty-two pairs of GC and their paired adjacent normal tissues (*n* = 22) were measured by real-time PCR (Paired *t*-test, ^∗^*P* < 0.05; ^∗∗^*P* < 0.01; ^∗∗∗^*P* < 0.001; ^****^P < 0.0001).

We analyzed the network of potential biological functions associated with the 11 pyroptosis-related regulators by using the STRING platform (Figure 1A). The regulators focused predominantly on the regulation of immune response and pyroptosis. At the genetic level, 59 of the 433 samples (about 13.63%) showed pyroptosis-related regulator mutations. Of these, *CASP5* showed the highest frequency of mutations. We did not identify any *GSDME* mutations in any of the GC samples (Figure 1B). We also found CNVs in 7 of the 11 pyroptosis-related regulators in GSE62717; these were common changes and most were concentrated on copy number amplification (Figure 1C). We identified the alterations of the 7 regulators featuring CNVs on the chromosome (Figure 1D). At the expression level, these 11 regulators were able to help us distinguish normal samples from tumor samples in GC patients (Figure 1E). Compared with normal samples, except for *GSDME* and *GZMA*, the remaining regulators all showed increases in GC samples (Figure 1F and Supplementary Figure 2A). To more effectively verify our findings, we tested the mRNA levels of 11 pyroptosis-related genes in 22 pairs of tumor tissues and normal adjacent tissue samples gathered from our hospital. The same finding was that these regulators were up-regulated in tumors (Figure 1G). In order to better explore the possible relationship between the genetic level and the expression level of pyroptosis-related regulators, we combined the CNV pattern of TCGA-STAD and its expression level for joint analysis. Interestingly, we found that the expression levels of *CASP1*, *CASP3*, *CASP4*, *CASP5*, and *CASP8*, all increased with increases in copy number; a similar relationship was identified for *GSDMD* (Supplementary Figures 2B–G). We hypothesized that changes in CNV may be an important factor that can lead to abnormal gene expression. Our analysis showed that the expression levels of pyroptosis-related regulators were related to GC, thus suggesting they may reflect different traits in patients.

### Identification of a GC Classification Pattern Mediated by 11 Pyroptosis-Related Regulators

**FIGURE 2:**
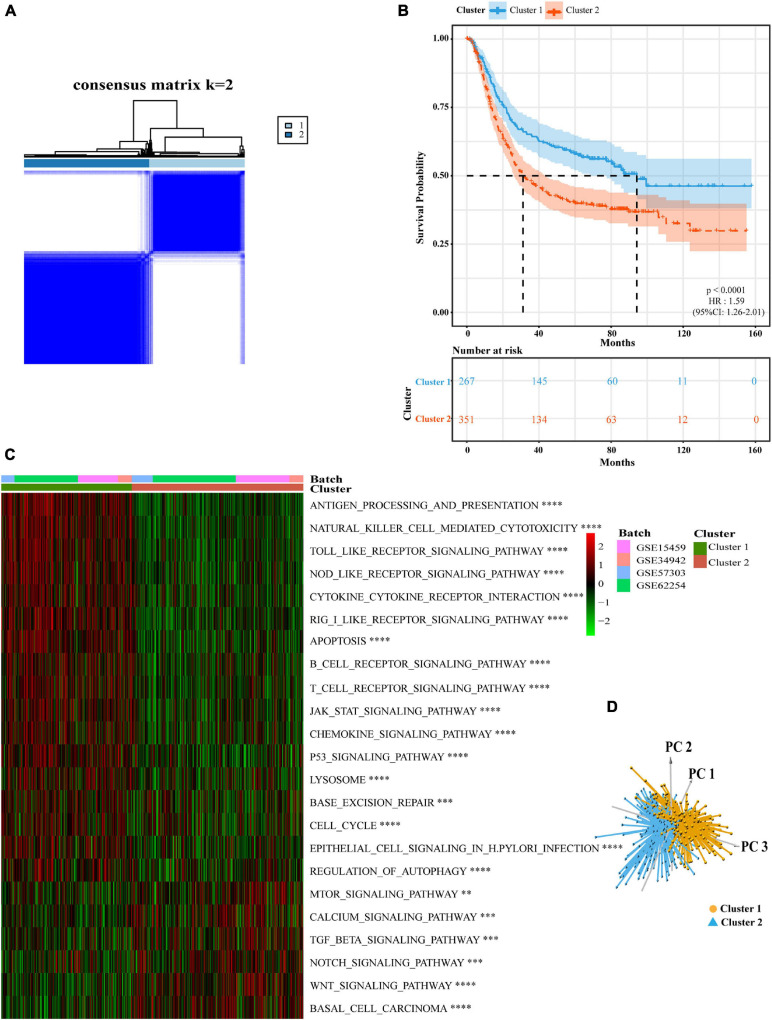
Subgroups of gastric cancer related by pyroptosis-related regulators. **(A)** The consensus score matrix of all samples when *k* = 2 in GEO cohorts (GSE15459, GSE34942, GSE57303, and GSE62254). Two samples were more likely to be grouped into the same cluster when there was a higher consensus score between them in different iterations. **(B)** OS curves for the two pyroptosis-related clusters based on 618 patients with gastric cancer from four GEO cohorts (GSE15459, GSE34942, GSE57303, and GSE62254) (Log-rank test, *p* < 0.0001). OS, Overall survival. **(C)** The heatmap was used to visualize biological processes analyzed by GSVA which showed the active biological pathways in distinct pyroptosis-related clusters (Bayes moderation, ^∗∗^*P* < 0.01; ^∗∗∗^*P* < 0.001; ^****^P < 0.0001). **(D)** Principal component analysis for the expression of pyroptosis-related regulators to distinguish cluster 1 (*n* = 267) from cluster 2 (*n* = 351) in GEO cohorts (GSE15459, GSE34942, GSE57303, and GSE62254).

We created a queue using four GEO datasets from the same platform along with OS data and clinical information. Based on the expression levels of 11 pyroptosis-related regulators, we identified two different regulation patterns by using the unsupervised clustering method, including 267 cases in pyroptosis-related cluster 1 and 351 cases in pyroptosis-related cluster 2 (Figure 2A, Supplementary Table 2, and Supplementary Figures 3A–I). The survival advantage of cluster 1 was higher than that of cluster 2 (Figure 2B). To explore the differences in biological behavior between these two patterns, we performed GSVA enrichment analysis (Figure 2C and Supplementary Table 3). Cluster 1 showed enrichment in terms of pathways associated with immune activation, including antigen processing and presentation, TOLL, NOD, and RIG I-like receptor signaling pathways, B cells and activated T-cell receptor signaling pathways, the activated JAK-STAT signaling pathway, base excision repair, and *H. pylori* infection resistance. Pyroptosis is known to be converted from apoptosis with different bacterial or viral infections (Rogers et al., 2017; Zhang Z. et al., 2020; Zhou et al., 2020). Therefore, cluster 1 was also significantly enriched in the apoptotic pathway. Cluster 2 showed enrichment in carcinogenic activation pathways, such as the mTOR, TGFβ, NOTCH, and WNT signaling pathways. Subsequently, we also confirmed that the two regulatory patterns could be distinguished by the application of the 11 pyroptosis-related regulators (Figure 2D).

### Differences in TME Infiltration and Clinical Characteristics Between Two Pyroptosis-Related Subtypes

**FIGURE 3:**
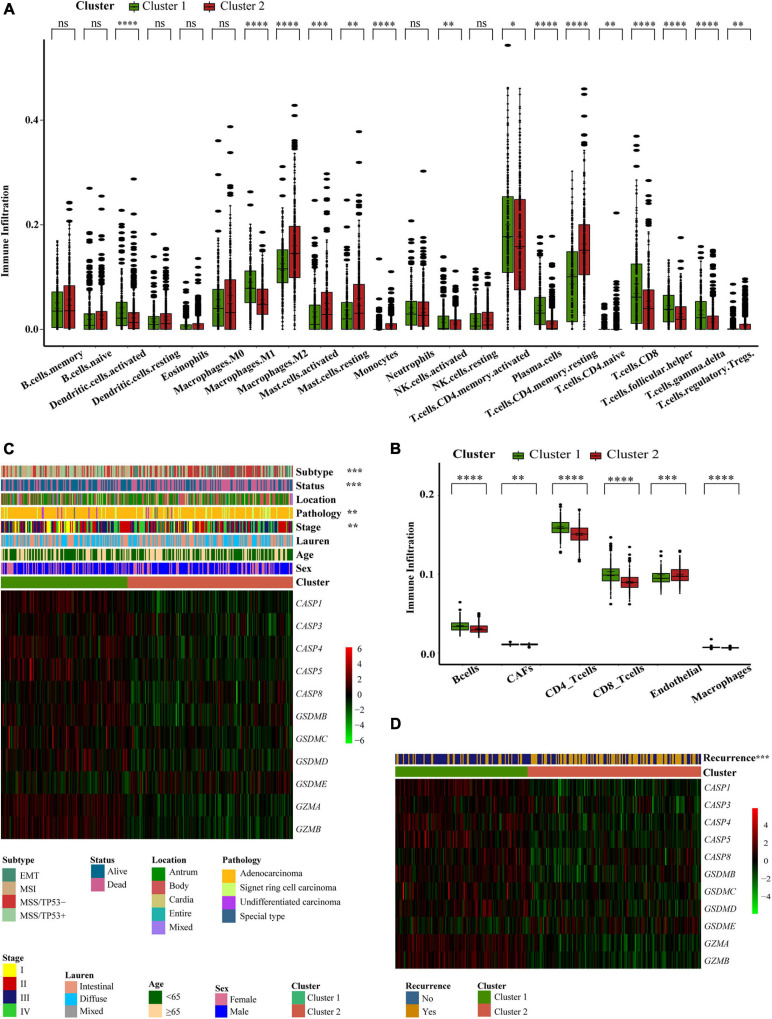
Different pyroptosis-related clusters showed diverse clinical features and TME cell infiltration. **(A,B)** The abundance of every type of TME infiltrating cells between the two pyroptosis-related clusters analyzed, respectively, by CIBERSORTx and EPIC in GEO cohorts (GSE15459, GSE34942, GSE57303, and GSE62254) (Wilcox test, ^∗^*P* < 0.05; ^∗∗^*P* < 0.01; ^∗∗∗^*P* < 0⋅001; ^****^P < 0.0001; ns, not statistically significant). **(C)** Consensus clustering of differential expression genes between the two pyroptosis-related clusters in GSE62254 cohort. Columns of the heatmap represented 300 gastric cancer samples (Chi-square test or Fisher’s exact test, ^∗∗^*P* < 0.01; ^∗∗∗^*P* < 0.001). **(D)** Unsupervised clustering of differential expression genes between the two pyroptosis-related clusters in GSE62254 cohort. Columns of the heatmap represented 282 gastric cancer patients with recurrence records (Chi-square test, ^∗∗∗^*P* < 0.001).

Next, we analyzed cell infiltration data and found that activated innate immune cell infiltration was abundant in cluster 1, including the presence of dendritic cells, M1 macrophages, B cells, along with activated CD 4 and CD 8 T cells, thus conferring a significant survival advantage. Cluster 2 was enriched with endothelial cells, mast cells, M2 macrophages, and resting T4 memory cells (Figures 3A,B). Although some tumor tissues possessed a large number of immune cells, these immune cells could not penetrate the tumor but were forced to stay in the surrounding matrix. The activation of the matrix in a tumor microenvironment was therefore considered to be immunosuppressive (Chen and Mellman, 2017). In the present analysis, the ESTIMATE score also showed that the immune score of cluster 1 was higher than that of cluster 2 (Supplementary Figure 3J). We found that these two regulatory patterns had completely different TME cell infiltration characteristics. Cluster 1 was classified as an immune-inflamed phenotype while cluster 2 was classified as an immune-excluded phenotype (Chen and Mellman, 2017). We also found that the proportion of TME cell types in the different clusters was different while the composition types were the same (Supplementary Figure 3K). These suggested that although these regulatory patterns did not increase or decrease the types of immune infiltrating cells, it was likely to change their proportion and thus change the TME.

To further explore the clinical manifestations of these two different clusters, we focused on the ACRG cohort which represents the most comprehensive study of clinical information relating to our 618 patients (Figure 3C). Most regulators were expressed at high levels in cluster 1. Patients with EMT molecular subtypes (Cristescu et al., 2015) were prominent in cluster 2, while patients with MSI subtypes were classified into cluster 1. Patient survival status also corresponded well to the survival advantages described in our previous research. Compared with other pathological types of GC, patients with signet ring cell carcinoma were mainly associated with the cluster 2 pattern. Moreover, we noticed that patients with early GC were associated with a cluster 1 regulatory pattern and that patients with advanced GC were mainly associated with the cluster 2 regulatory pattern. This explains why the cluster 1 regulatory pattern was associated with a better survival advantage. We conducted statistical analyses on 282 patients that possessed recurrence data in the ACRG cohort. The cluster 2 pattern was associated with a greater number of recurrence cases (Figure 3D). These findings showed that different pyroptosis-related patterns represented different GC features and had different TME statuses.

### Development and Validation of a Gene Signature Based on Pyroptosis-Related Clusters

**FIGURE 4:**
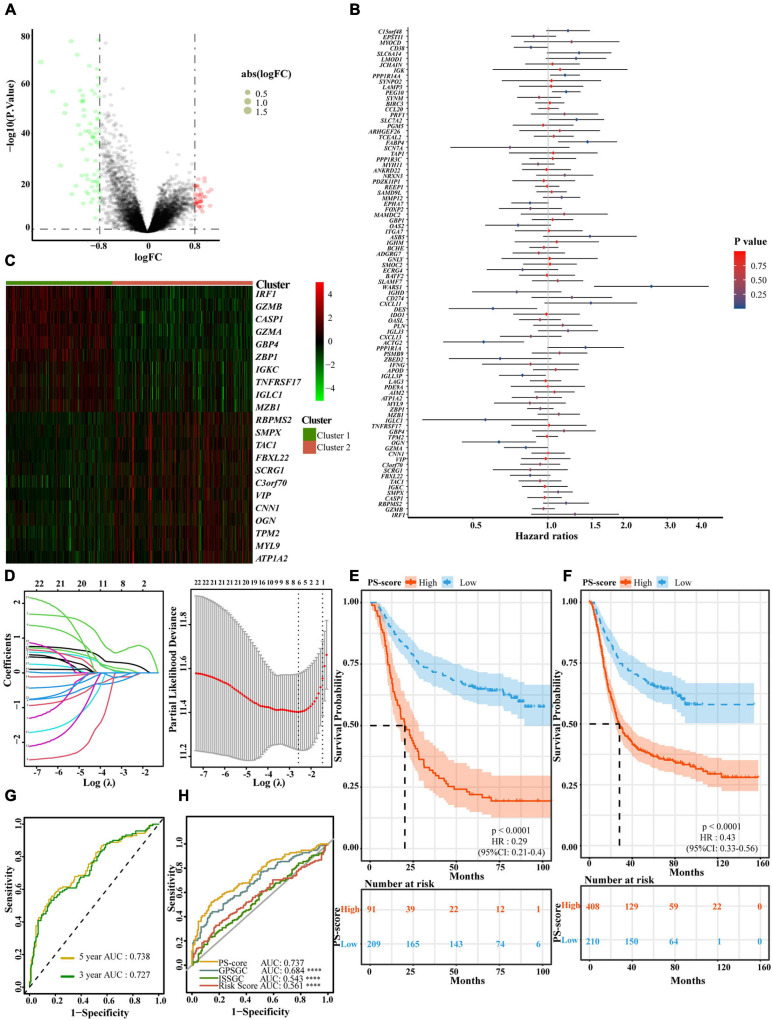
Generation of a gene expression signature to predict patient survival based on pyroptosis-related clusters. **(A)** An overview of the differential gene expression between the two pyroptosis-related clusters in GEO cohorts (GSE15459, GSE34942, GSE57303, and GSE62254). **(B)** Multivariate Cox regression analyses of OS in GEO cohorts (GSE15459, GSE34942, GSE57303, and GSE62254). The *p*-values were obtained by multivariate Cox regression. No Cox regression assumptions were violated assessed using the SPSS 26 software. **(C)** Unsupervised clustering of independent prognostic genes in GEO cohorts (GSE15459, GSE34942, GSE57303, and GSE62254). Columns of the heatmap represented 618 patients with gastric cancer. **(D)** In the LASSO-Cox model of GSE62254, the minimum standard was adopted to obtain the value of the super parameter λ by 10-fold cross-validation. The λ value was confirmed as 0.07558 where the optimal lambda resulted in 6 non-zero coefficients. **(E)** OS curves for the different PS-score subgroups with the cut-off value 1,258 about 300 patients with gastric cancer from GSE62254 cohort (Log-rank test, *p* < 0.0001). **(F)** OS curves for the different PS-score subgroups with the cut-off value 1⋅258 among 618 gastric cancer samples from four GEO cohorts (GSE15459, GSE34942, GSE57303, and GSE62254) (Log-rank test, *p* < 0.0001). **(G)** The time-dependent receiver operating characteristic (ROC) analysis of the PS-score. The area under the curve (AUC) was 0.727, 0.738 at 3 years, and 5 years, respectively, in GSE62254 cohort. **(H)** ROC curves about PS-score ISSGC Score, Risk Score, and GPSGC Score in GSE62254 cohort (Mann Whitney tests; compared with PS-score, ^****^P < 0.0001).

To better apply these subtypes to the clinical treatment of GC and determine a specific score for every patient, we next explored differences between the two patterns and determined a specific gene signature. We also quantified the gene signature so that it could be applied to the diagnosis and treatment of each patient. First, we identified 113 DEGs with an absolute value of Log2 FC < 0⋅8 and *p* < 0⋅05 associated with the two different regulatory patterns (Figure 4A and Supplementary Table 4). Next, univariate and multivariate Cox regression analysis identified 22 genes that could be used as an independent prognostic signature (Figure 4B and Supplementary Tables 5, 6). Ten genes, identified as the most immune response-related genes, appeared in cluster 1 while 12 genes encoding cancer occurrence proteins tended to be more prevalent with the cluster 2 pattern (Figure 4C). To build a model that would be able to quantify each patient, six of the 22 DEGs were retained by application of LASSO-Cox regression model with a minimum of λ. We used these to build a pyroptosis-related signature score which we named the “PS-score” (Figure 4D and Table 1). Next, we attempted to further determine the value of PS-score by predicting the prognosis of patients. We divided patients into high and low PS-score groups with the best cut-off value of 1⋅258. We found that the low group had a clear survival advantage over the high group (Supplementary Figures 4A,B). To prove the universal indicative value of the PS-score, we also verified this score in more cohorts and obtained the same results (Figures 4E,F). We further proved that the PS-score was a good indicator for the 3-year survival and 5-year survival of GC patients (Figure 4G and Supplementary Figure 4C). Because the prognostic labels of GC have been discussed extensively in recent years and play an important role in early diagnosis and treatment, we also compared several other rigorous prognostic models to evaluate the important function of the PS-score for evaluating prognosis. The analysis showed that the PS-score was better than other models for predicting the prognosis of GC patients (Cho et al., 2011; Cai et al., 2020 Wang et al., 2020: Figure 4H). Therefore, we included PS-score as an effective indicator and other clinical characteristics, into Cox regression analysis and found that PS-score and stage were both two factors that independently affected the prognosis of GC patients (Table 2). Interestingly, we found that the combination of PS-score and GC stage could better predict the survival of patients (Supplementary Figure 4D). These findings indicated that the PS-score was a promising potential evaluation indicator for the prognosis of GC patients.

**TABLE 1:** Pyroptosis-related signature score (PS-score).

Gene	Power
GZMB	–0.10731
RBPMS2	0.729766
CASP1	–0.12987
TAC1	0.259529
TPM2	0.081455
GBP4	–0.01268
lambda.min = 0.07557829	

**TABLE 2:** Analysis of factors affecting the prognosis of patients with gastric cancer.

	Number of patients	Proportion (%)	Univariate Cox analysis		Multivariate Cox analysis	
			HR (95% CI for HR)	*p*.value	HR (95% CI for HR)	*p*.value
**Sex**				0.56	–	–
Male	199	66.30	1.00			
Female	101	33.70	1.11 (0.79–1.55)			
**Age**				0.09	–	–
<65	172	57.30	1.00			
≥65	128	42.70	1.32 (0.96–1.81)			
**Lauren**				0.003		0.38
Diffuse	135	45.00	1.00		1.00	
Intestinal	146	48.70	0.60 (0.43–0.84)		0.83 (0.57–1.23)	
Mixed	19	6.30	1.27 (0.71–2.28)		1.27 (0.68–2.37)	
**Stage**				<0.0001		<0.0001
I	31	10.30	1.00		1.00	
II	97	32.30	2.00 (0.78–5.16)		1.66 (0.64–4.33)	
III	95	31.70	4.21 (1.68–10.54)		3.01 (1.18–7.71)	
IV	77	25.70	10.09 (4.05–25.14)		7.44 (2.92–19.00)	
**Pathology**				0.001		0.112
Adenocarcinoma	250	83.30	1.00		1.00	
Signet ring cell carcinoma	37	12.30	2.38 (1.59–3.56)		1.57 (0.94–2.64)	
Undifferentiated carcinoma	9	3.00	0.72 (0.23–2.28)		0.77 (0.24–2.47)	
Special type	4	1.30	2.43 (0.77–7.67)		3.37 (1.03–11.05)	
**Location**				0.133	–	–
Cardia	30	10.00	1.00			
Body	107	35.70	0.64 (0.38–1.07)			
Antrum	150	50.00	0.60 (0.37–0.99)			
Entire	4	1.30	1.90 (0.65–5.57)			
Mixed	9	3.00	0.53 (0.18–1.55)			
**Subtype**				<0.0001		0.176
EMT	46	15.30	1.00		1.00	
MSS/TP53-	107	35.70	0.64 (0.42–0.98)		1.76 (1.04–2.98)	
MSS/TP53+	79	26.30	0.50 (0.32–0.80)		1.78 (0.98–3.24)	
MSI	68	22.70	0.33 (0.20–0.57)		1.64 (0.84–3.23)	
**PS-score**				<0.0001		<0.0001
Low	209	69.70	1.00		1.00	
High	91	30.30	3.41 (2.47–4.71)		3.15 (2.14–4.64)	

### Low PS-Scores Identified the Alleviation of Clinical Characteristics and Immune Pathway Activation

**FIGURE 5:**
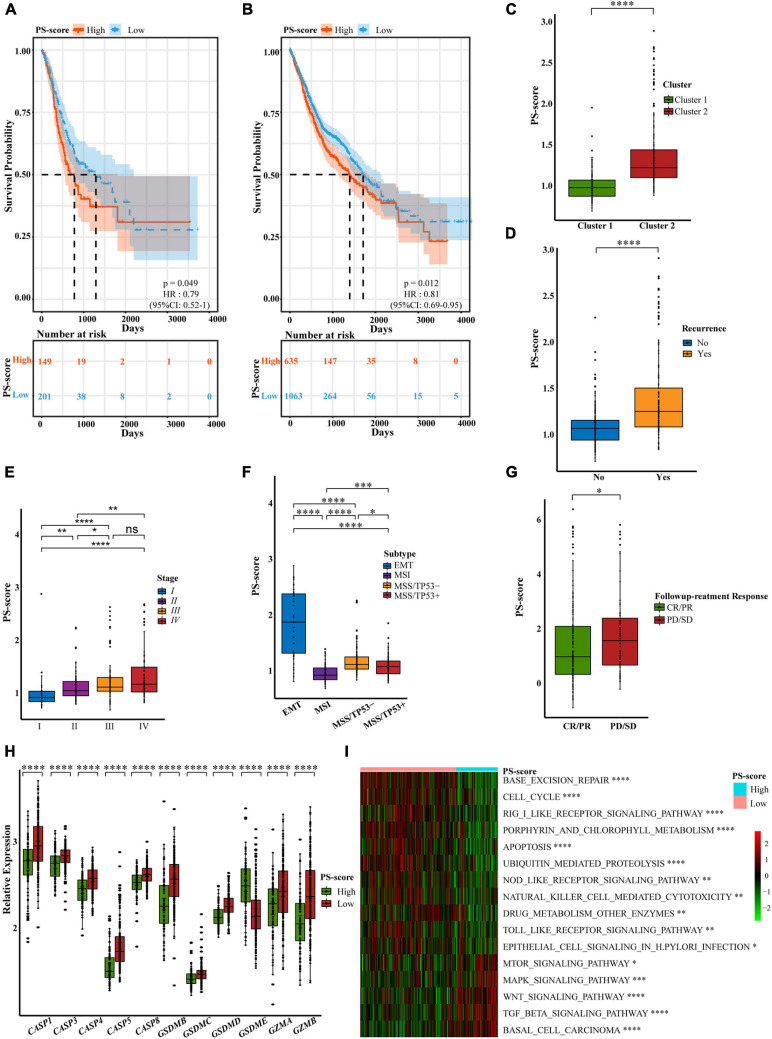
Characteristics of the PS-score scoring model. **(A)** OS curves for the PS-score with the cut-off value 1,258 of gastric cancer samples in TCGA-STAD cohort (Log-rank test, *p* = 0.049). **(B)** OS curves for the PS-score with the cut-off value 1,258 of 1,698 gastrointestinal cancer patients in TCGA cohorts (CHOL, COAD, ESCA, LIHC, PAAD, READ, and STAD) (Log-rank test, *p* = 0.012). **(C–G)** PS-score in different clinical trait constituents including pyroptosis-related cluster, recurrence status, TNM staging, ACRG subtype in GSE62254 cohort, and followup treatment response in TCGA-STAD (Wilcox test, ^∗^*P* < 0.05; ^∗∗^*P* < 0.01; ^∗∗∗^*P* < 0.001; ^****^P < 0.0001; ns, not statistically significant). **(H)** Differential expression of pyroptosis-related regulators in low PS-score subgroup (*n* = 209) and high PS-score subgroup (*n* = 91) of GSE62254 cohort (Wilcox test, ^****^P < 0.0001). **(I)** Visualization of biological processes analyzed by GSVA in distinct PS-score subgroups (Bayes moderation, ^∗^*P* < 0.05; ^∗∗^*P* < 0.01; ^****^P < 0.001; ^****^P < 0.0001).

We also found that the high-score group in the TCGA-STAD cohort predicted poor survival characteristics with the same cut-off value (Figure 5A). And we were pleasantly surprised to find that PS-score not only predicted the overall survival of patients with GC but also could predict the disease-specific survival of patients specifically (Supplementary Figure 4E). Subsequently, to verify the universal applicability of PS-score for all digestive tract cancers, we collected data from seven digestive tract cancer samples including gastric cancer and divided these into two groups with the same cut-off value. We were surprised to find that the low-score group also showed a survival advantage (Figure 5B). This implied that the PS-score may have an important value as a prognostic indicator not only in GC but also in other forms of gastrointestinal cancer.

To determine the specificity of the PS-score in patients with different clinical manifestations, we analyzed the relationship between clinical traits and PS-score in the ACRG cohort. We found that the cluster 1 pattern, with an obvious survival advantage, presented with obviously low scores; this was consistent with our previous research (Figure 5C). Patients with high scores also experienced more relapses (Figure 5D). We were surprised to find that with an increase in patient survival stage, the PS-score showed a gradually increasing trend, thus showing that the score for patients with advanced gastric cancer was higher than that for patients with early gastric cancer (Figure 5E). This suggested that PS-score had the potential as a clinical index to quantify the survival risk of GC patients. Similarly, the EMT subtype with a poor response to treatment (Cristescu et al., 2015) also exhibited high scores (Figure 5F). We also found that different Lauren classifications and pathological types showed completely different PS-score values (Supplementary Figures 4F,G). Compared with other pathological types, patients with signet ring cell carcinoma which were mainly associated with the cluster 2 pattern, had high PS-scores. We also found that second/third-line treatment was the commonly used strategy in the clinic. However, this system lacks a suitable measurement standard. A low PS-score indicated a good response to followup treatment, thus indicating that PS-score may represent a good marker for GC treatment (Figure 5G). As the main cause of gastric cancer, the screening of *H. pylori* has significantly reduced the prevalence of GC (Smyth et al., 2020). Therefore, we specifically analyzed the relationship between *H. pylori* infection and PS-score and found that patients with positive infection may have higher scores (Supplementary Figure 4H). Although the limitation of the sample size made the statistical significance less obvious, we still observed a trend consistent with the previous results. This gave us a hint, but more verification is still needed.

In addition to different clinical phenotypes, the expression of different pyroptosis-related regulators in patients would also affect the specificity of the PS-score. We found that regardless of whether we used the ACRG cohort or the TCGA-STAD cohort, except for *GSDME*, the expression levels of other regulators were significantly reduced in the high-score group (Figure 5H and Supplementary Figure 4I). *GSDME* was generally expressed at higher levels in the high-score group; this may be related to the cytokine release syndrome (CRS) induced by patients with high *GSMDE* expression during treatment (Liu et al., 2020a). CRS was characterized by fever, hypotension, and respiratory insufficiency associated with elevated serum cytokines (Davila et al., 2014), which was positively correlated with *GSDME* expression level (Liu et al., 2020a). Therefore, treatments for *GSDME* should be used with caution to avoid undesirable side effects (Ibrahim et al., 2021). Although *GSDME* is considered to be a probable tumor suppressor gene (Wang et al., 2017), its precise function needs to be explored further. We were unable to judge the prognosis of patients by considering only the level of *GSDME* expression. Similarly, it would be unscientific to predict the survival ability of GC patients solely by considering the expression of pyroptosis-related regulators. Our research aimed to create a quantitative pyroptosis-related model to comprehensively predict prognosis and the value of treatment. Based on the specificity data supporting the PS-score, we explored the pathway enrichment associated with the PS-score to identify the internal mechanisms involved. We found that the low-score group was significantly enriched in inflammatory signaling pathways. The PS-score showed a positive response to base damage repair and response to *H. pylori* infection and was also related to ubiquitination modification and drug metabolism regulation. However, the high-score group was mainly enriched in signal pathways related to cancer development (Figure 5I and Supplementary Table 7). These findings showed that PS-score exerted specificity in different patients associated with different patterns of signaling pathway activation and could be used to evaluate certain clinical characteristics and therapeutic effects in GC patients.

### The PS-Score Could Predict Prognosis in Clinical Scenarios and Represent TME Differences

**FIGURE 6:**
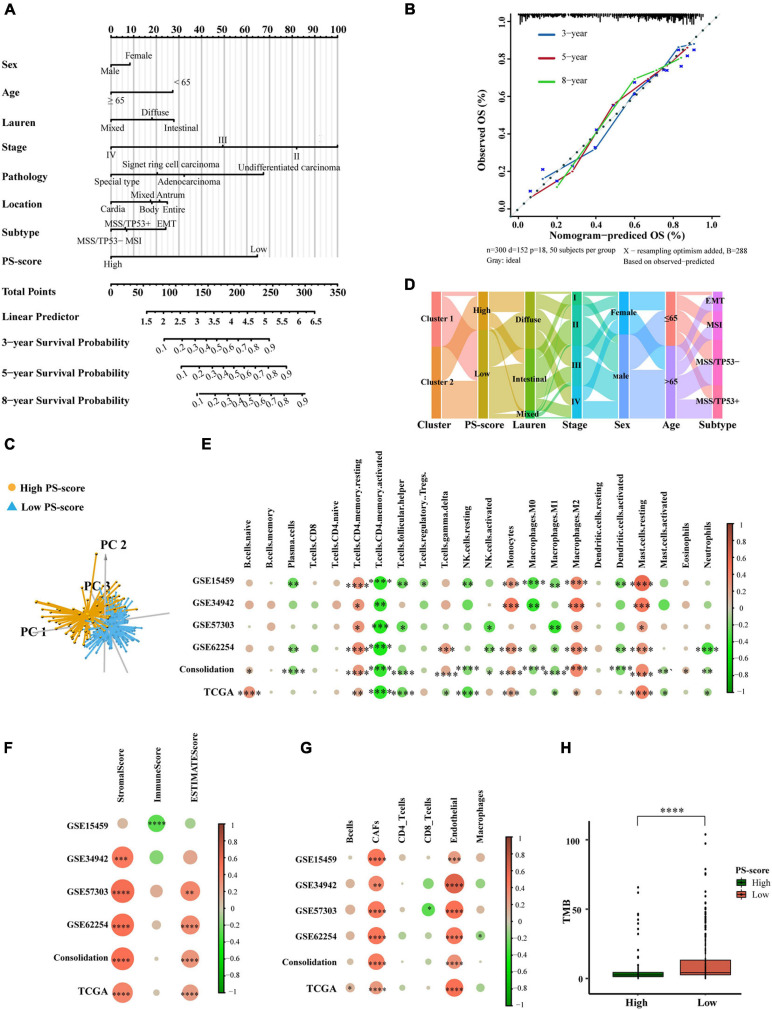
The clinical application value of the PS-score scoring model and TME of different PS-score subgroups. **(A)** Nomogram for predicting 3, 5, and 8 years overall survival for gastric cancer patients in GSE62254 cohort. **(B)** Calibration curves of nomograms in terms of the agreement between predicted and observed 3, 5, and 8 years of outcomes in GSE62254 cohort. **(C)** Principal component analysis for the expression of the PS-score signature genes to distinguish low and high PS-score subgroups in GSE62254 cohort. **(D)** Alluvial diagram showing the changes of pyroptosis-related clusters, PS-score, Lauren type, TMN staging, sex, age, and ACRG subtypes in GSE62254 cohort. **(E–G)** The correlation between every type of TME infiltrating cells and PS-score analyzed, respectively, by CIBERSORTx, ESTIMATE score, and EPIC in cohorts (GSE15459, GSE34942, GSE57303, GSE62254, and TCGA-STAD; Consolidation was the cohort consolidating four GEO cohorts) (Spearman test, ^∗^*P* < 0.05; ^∗∗^*P* < 0.01; ^∗∗∗^*P* < 0.001; ^****^P < 0.0001). Sizes of circles represented relevant correlation coefficients. **(H)** Tumor burden (TMB) of low PS-score subgroup (*n* = 215) and high PS-score subgroup (*n* = 153) in TCGA-STAD (Wilcox test, ^****^P < 0.0001).

Given the importance of the PS-score in predicting the prognosis of GC patients, we next attempted to explore its value for clinical application. We constructed a nomograph featuring seven clinical features that were easily accessible and generally believed to have a certain impact on the prognosis of GC and the ability of the PS-score to predict the survival rates of GC patients at 3, 5, and 8 years (Figure 6A). A C-index of 0⋅764 indicated that the nomogram had a good predictive value (Figure 6B). When we used the PS-score as a separate indicator to distinguish patients with GC, we found the 300 patients of the ACRG cohort were divided into a high group and a low group (Figure 6C). An alluvial diagram was used to visualize the changes in patient characteristics (Figure 6D).

Based on these results, we found that PS-score could play an important role in clinical prediction. Next, we investigated whether PS-score would have a guiding value for clinical treatment, especially immunotherapy. We analyzed the infiltration of TME cells with different PS-scores (Figures 6E–G). Immune activation-related cells, such as activated T cells, NK cells, M1 macrophages, dendritic cells, neutrophils, showed significant negative correlations with PS-score. The higher scores were closely related to resting memory cells, monocytes, mast cells, cancer-associated fibroblasts, and endothelial cells. The immunescore from ESTIMATE analysis decreased as the PS-score increased, while the stromalscore showed the opposite effect. These data showed that the low-score group had a stronger immune response than the high-score group. The differences in TME cells may be the main reason for the heterogeneity of PS-score. Tumors that could attract more T cell infiltration are referred to as “hot tumors” and are more sensitive to immunotherapy and showed better immunotherapy effects (Li et al., 2018). Clinical trials and preclinical studies have also revealed that patients with higher somatic tumor mutational burden (TMB), when treated with immune checkpoint blockade therapy, were associated with enhanced responses, long-term survival, and lasting clinical benefits (Zhang B. et al., 2020). Fortunately, we explored the TMB of different PS-scores and found that the lower group had a higher TMB, which suggested a better immunotherapy response (Figure 6H). These results proved that PS-score may have an application value for predicting the prognosis of GC patients, and would reflect the response of immunotherapy to a certain extent.

### The Role of PS-Score in Anti-PD1/PD-L1 Immunotherapy

**FIGURE 7:**
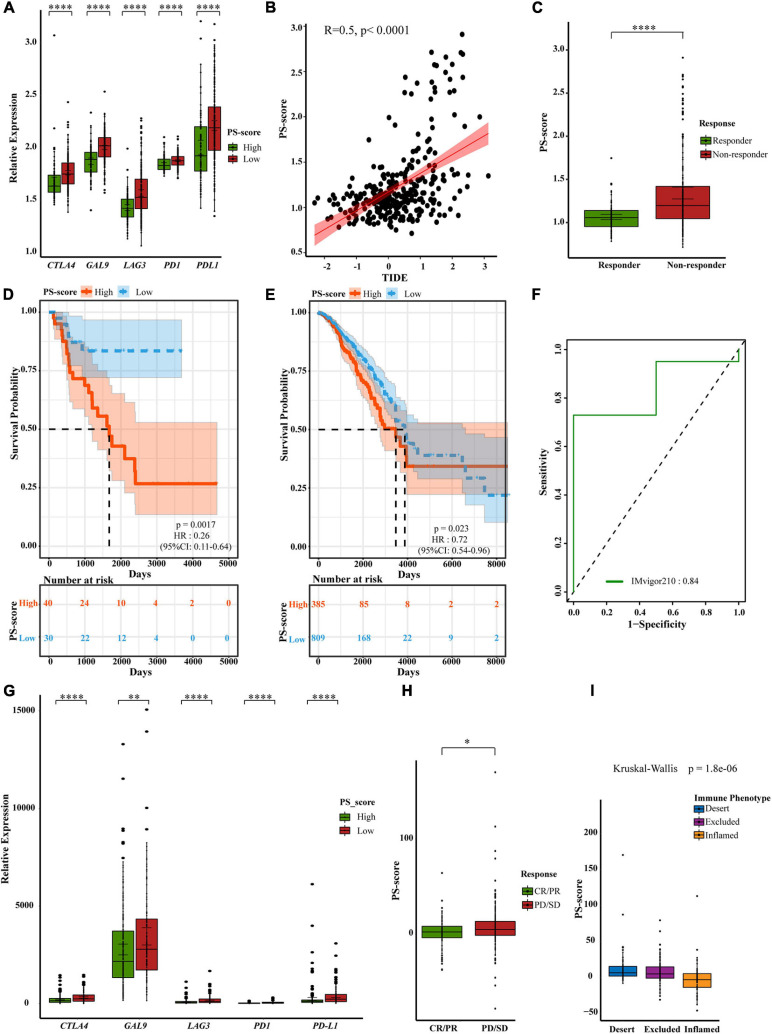
A powerful role of the PS-score scoring model in PD1/PD-L1 immunotherapy. **(A)** Differential expression of immune checkpoint genes in low PS-score subgroup (*n* = 209) and high PS-score subgroup (*n* = 91) of GSE62254 cohort (Wilcox test, ^****^P < 0⋅0001). **(B)** The relationship between TIDE and PS-score in GSE62254 cohort (Spearman test, *p* < 0⋅0001). **(C)** Different PS-score in responder group (*n* = 112) and non-responder group (*n* = 188) in GSE62254 cohort (Wilcox test, ^****^P < 0.00001). **(D,E)** OS curves for the PS-score with the cut-off value 1,258 of samples in TCGA-ACC cohort (Log-rank test, *p* = 0.0017) and TCGA-BRCA cohort (Log-rank test, *p* = 0.023). **(F)** ROC curves about PS-score in IMvigor210. **(G)** Differential expression of immune checkpoint genes in low PS-score subgroup (*n* = 126) and high PS-score subgroup (*n* = 222) of IMvigor210 cohort (Wilcox test, ^∗∗^*P* < 0.01; ^****^P < 0.0001). **(H)**. Different PS-score in CR/PR group (*n* = 68) and SD/PD group (*n* = 230) in IMvigor210 cohort (Wilcox test, ^∗^*P* < 0.05). SD, stable disease; PD, progressive disease; CR, complete response; PR, partial response. **(I)** Different PS-score in IMvigor210 cohort’s immune phenotypes (Kruskal-Wallis test, *P* = 1.8e-06).

Previous studies have shown that PS-score may suggest the effect of immunotherapy. There is also evidence that patients with a high TMB status show long-lasting clinical responses to anti-*PD1*/*PD-L1* immunotherapy (Zhang B. et al., 2020). Recent studies have shown that there was a clear connection between the expression of *PD-L1* and pyroptosis. Therefore, we speculated that there may be a connection between PS-score and immunotherapy (Hou et al., 2020). We first checked the expression changes of immune checkpoints. We compared the differences in the expression levels of immune checkpoint genes in the ACRG cohort and the TCGA-STAD cohort (Figure 7A and Supplementary Figure 5A). We found that the low-score group showed higher expression levels of immune checkpoint genes, thus suggesting a better response to immunotherapy. Tumor Immune Dysfunction and Exclusion (TIDE) is a computational framework to model two primary mechanisms of tumor immune evasion which can provide predicted results about immunotherapy (Jiang P. et al., 2018; Chen et al., 2021). High TIDE may predict patients with suppressive cells inhibiting T cell infiltration as non-responders. To better illustrate the predictive power of the PS-score for immunotherapy, we applicated TIDE in ACRG cohort. We were pleasantly surprised to find a positive correlation between TIDE and PS-score (Figure 7B). Furthermore, predicted responses suggested that PS-score may be a good predictor of immunotherapy in GC (Figure 7C). As we all know, “hot tumor” is more sensitive to immunotherapy (Galon and Bruni, 2019). We had also verified the higher PS-score represented worse prognostics in “hot tumor” such as breast cancer and kidney cancer (Figures 7D,E). Due to the lack of published data on anti-*PD1*/*PD-L1* immunotherapy in GC, we investigated published three datasets: IMvigor210, NCT01358721, and GSE78220 (Choueiri et al., 2016; Hugo et al., 2016; Mariathasan et al., 2018). Interestingly, when we analyzed the prognosis of patients with these three cancers, we found that PS-score could be a good predictor for melanoma patients and metastatic renal cell carcinoma patients with 1-year survival, and metastatic urothelial cancer patients’ 2-year survival rate (Figure 7F and Supplementary Figures 5B,C). So we guessed that PS-score had a connection with immunotherapy in other cancers. Then we detected changes in the expression of immune checkpoints presented by different PS-scores and also found that the low-score group had higher expression (Figure 7G), thus suggesting that PS-score may be an important indicative index that plays the same important role in other cancers. As we hypothesized, we found that patients who responded to immunotherapy also showed a lower PS-score (Figure 7H and Supplementary Figures 5D,E). In metastatic urothelial cancer, different immunological subtypes may lead to a completely different therapeutic response. We also found that they represented different levels of PS-score (Figure 7I). These results explained the potential value of PS-score in immunotherapy, and to a certain extent proved that PS-score may be used as a predictor of immunotherapy responses.

## Discussion

Chronic infection of the gastric mucosa leads to the gradual development of atrophic gastritis and intestinal metaplasia, thus promoting the progression of GC (Smyth et al., 2020). Molecular signatures associated with distinct clinical outcomes have been delineated in various solid tumors to improve clinical management through the development of personalized medicine (Sorlie et al., 2001; Cho et al., 2011; Higgins and Baselga, 2011; Li et al., 2013; Roepman et al., 2014). Therefore, we need to explore the changes in the status and mechanisms of GC cells associated with the immune environment to facilitate treatment.

Pyroptosis occurs in cells infected by pathogens, as an embodiment of programmed cell death, thus inducing the body’s inflammatory response (Bedoui et al., 2020). Under the stimulation of pathogens, apoptosis can thus be converted into pyroptosis. Pyroptosis plays various roles in many cancers. It has the effect of inhibiting tumor growth in colorectal cancer, liver cancer, and skin cancer (Zaki et al., 2010; Ellis et al., 2011; Ma et al., 2016), but a two-way effect in breast cancer (Chen et al., 2012). So we cannot judge the prognostic value of GC based on the expression of several gasdermins alone. Therefore, we explored all the pathways directly related to pyroptosis and explored a prognostic signature by analyzing the influence of the involved pathways on the tumor microenvironment. Gasdermins blocker is under development, but there is insufficient evidence to support it. Our signature provides potential targets for targeted therapy of pyroptosis, especially *CASP1* and *GZMB*. At present, pyroptosis has been considered for use in anti-tumor therapy, and our research suggests that pyroptosis combined with immunotherapy to improve the prognosis of patients may be an effective treatment direction.

The classification of samples based on predefined gene expression characteristics is a proven method (Cristescu et al., 2015). Our subtyping strategy drew on this method and classified GC patients based on the expression of pyroptosis-related regulators. We showed that the expression of these regulators was completely different when compared between the two clusters due to various heterogeneities. These regulators were also significantly associated with different survival risks. Our analysis culminated in several consensuses: (1) most pyroptosis-related regulators showed high expression levels in cluster 1; (2) cluster 2 was a separate subtype which showed a worse prognosis; (3) cluster 1 was determined to be an immune-inflamed phenotype and cluster 2 was determined as an immune-excluded phenotype; and (4) collectively considering clinical information and RNA data was more likely to reflect the cellular phenotypes.

Clinical trials have tested anti-tumor molecular targeted drugs in all GC types regardless of the molecular subtypes involved. For example, the expression of immune checkpoint molecules differs across different subtypes, therefore immunotherapy should be distinguished. To create a better clinical application value, we developed a scoring model (PS-score) to quantify the prognostic risk based on the two clusters. Our study provided strong evidence for the clinical management of GC. First, the PS-score takes into account the heterogeneity of patients. Second, this score can link pyroptosis and prognosis. Specifically, the PS-score featured both tumor suppressor genes and tumor-promoting genes and allocated these with different weightings. The PS-score included but was not limited to pyroptosis-related regulators such as *GZMB* and *CASP1*. Third, the PS-score represented patients with different clinical traits and was related to immunotherapy. A high PS-score showed worse clinical traits and a lower predicted survival time. TME cell infiltration data demonstrated that the PS-score holds important value for immunotherapy. More activated immune cell infiltration in patients with a low PS-score led to a better response to immunotherapy. Compared with TMB and *PD-L1* expression data, the PS-score is affordable and provides more informative outcomes. Fourth, the PS-score may also apply to other gastrointestinal tumors and immune-related tumors. Finally, compared with other models, the PS-score is directly focused on the death mode of GC cells. Researchers have investigated prognostic models of GC in hypoxia or under modified conditions of m6A already or have considered the immunoscore (Jiang Y. et al., 2018; Liu et al., 2020b; Zhang B. et al., 2020). Our study was more focused on the factors that directly caused tumor cell death and changed the tumor microenvironment. In this manner, our model is more valuable for facilitating treatment.

Viral and bacterial infections in the stomach can trigger downstream signaling pathways through pattern recognition receptors (PRRs) such as NLRs and TLRs. The activated caspases cleave the pyroptosis-related genes, causing cell dilation and death, and releasing mature inflammatory factors, especially IL-18 and IL-1. This process not only responds to infection but also alters the tumor microenvironment. More immune cell infiltration means more favorable immunotherapy. At the same time, the occurrence of pyroptosis, such as the increase of *GSDMD* splice, can inhibit MAPK, mTOR, and Wnt signaling pathways to various degrees. We speculate that it plays an anti-tumor role and improves prognosis mainly by inhibiting cell proliferation. In the process of chemotherapy, *GSDME* cleavage can promote the transformation of cells from apoptosis to pyroptosis and promotes TIL function, thus creating conditions for immunotherapy. *GSDMB* cleaved by *GZMA* also can convert apoptosis into pyroptosis, and IFN-γ promoted *GZMA*- or NK cell-induced pyroptosis in several target cells. High-level expression of *GSDMB* in cancer cells enhanced tumor clearance in a mouse model. These also mean that pyroptosis-related genes may be able to predict the prognosis and prompt immunotherapy.

Our study aimed was to classify patients with GC into subtypes, identify DEGs and build a prognostic model, and link pyroptosis with patient prognosis. Although we had performed multi-angle and multi-database verifications, this study still had limitations that need to be considered. The model created did not have a good predictive value in terms of all-time survival stages in patients undergoing immunotherapy. This may have been caused by the specificity of different cancers and requires more extensive research. Tumor heterogeneity is indeed a problem that cannot be ignored, and more targeted improvements for different types of tumors may be proposed with the development of pyroptosis-related researches. There is also a need for excavating or building more gastric cancer immunotherapy data and *H. pylori* infection data. The results of some single-cell sequencing should be able to explain the specific changes in the tumor microenvironment, which is also an aspect of our attention in the future. Moreover, our model should be validated further by performing both *in vitro* and *in vivo* experiments to better evaluate the relationship between the PS-score and pyroptosis of cells after infection. These have not only increased the challenges but also added hope for us to make us more motivated to continue digging.

## Data Availability Statement

The original contributions presented in the study are included in the article/Supplementary Material, further inquiries can be directed to the corresponding author/s.

## Ethics Statement

The studies involving human participants were reviewed and approved by the Committee of theSchool of Basic Medical Science, Cheeloo College of Medicine, Shandong University. The patients/participants provided their written informed consent to participate in this study.

## Author Contributions

JJ designed the study, obtained the funding, and supervised the study. WS, ZY, LZ, and FL organized the data. WS and ZY performed the analysis. YF checked the statistical method. JJ, WS, ZY, and YF prepared the figures. WS, ZY, YF, and LC wrote the manuscript. All authors contributed to the article and approved the submitted version.

## Conflict of Interest

The authors declare that the research was conducted in the absence of any commercial or financial relationships that could be construed as a potential conflict of interest.

## References

[B1] Bedoui S. Herold M. J. Strasser A. (2020). Emerging connectivity of programmed cell death pathways and its physiological implications. *Nat. Rev. Mol. Cell. Biol.* 21 678–695. 10.1038/s41580-020-0270-8 32873928

[B2] Begnami M. D. Fukuda E. Fregnani J. H. Nonogaki S. Montagnini A. L. da Costa W. L. Jr. (2011). Prognostic implications of altered human epidermal growth factor receptors (HERs) in gastric carcinomas: HER2 and HER3 are predictors of poor outcome. *J. Clin. Oncol.* 29 3030–3036. 10.1200/jco.2010.33.6313 21709195

[B3] Bray F. Ferlay J. Soerjomataram I. Siegel R. L. Torre L. A. Jemal A. (2018). Global cancer statistics 2018: GLOBOCAN estimates of incidence and mortality worldwide for 36 cancers in 185 countries. *CA Cancer J. Clin.* 68 394–424. 10.3322/caac.21492 30207593

[B4] Broz P. Pelegrin P. Shao F. (2020). The gasdermins, a protein family executing cell death and inflammation. *Nat. Rev. Immunol.* 20 143–157. 31690840 10.1038/s41577-019-0228-2

[B5] Cai W. Y. Dong Z. N. Fu X. T. Lin L. Y. Wang L. Ye G. D. (2020). Identification of a tumor microenvironment-relevant gene set-based prognostic signature and related therapy targets in gastric cancer. *Theranostics* 10 8633–8647. 10.7150/thno.47938 32754268 PMC7392024

[B6] Chen D. S. Mellman I. (2017). Elements of cancer immunity and the cancer-immune set point. *Nature* 541 321–330. 10.1038/nature21349 28102259

[B7] Chen L. C. Wang L. J. Tsang N. M. Ojcius D. M. Chen C. C. Ouyang C. N. (2012). Tumour inflammasome-derived IL-1beta recruits neutrophils and improves local recurrence-free survival in EBV-induced nasopharyngeal carcinoma. *EMBO Mol. Med.* 4 1276–1293. 10.1002/emmm.201201569 23065753 PMC3531603

[B8] Chen Y. Li Z. Y. Zhou G. Q. Sun Y. (2021). An immune-related gene prognostic index for head and neck squamous cell carcinoma. *Clin. Cancer Res.* 27 330–341. 10.1158/1078-0432.Ccr-20-2166 33097495

[B9] Cho J. Y. Lim J. Y. Cheong J. H. Park Y. Y. Yoon S. L. Kim S. M. (2011). Gene expression signature-based prognostic risk score in gastric cancer. *Clin. Cancer Res.* 17 1850–1857. 10.1158/1078-0432.Ccr-10-2180 21447720 PMC3078023

[B10] Choueiri T. K. Fishman M. N. Escudier B. McDermott D. F. Drake C. G. Kluger H. (2016). Immunomodulatory activity of nivolumab in metastatic renal cell carcinoma. *Clin. Cancer Res.* 22 5461–5471. 10.1158/1078-0432.Ccr-15-2839 27169994 PMC5106340

[B11] Cristescu R. Lee J. Nebozhyn M. Kim K. M. Ting J. C. Wong S. S. (2015). Molecular analysis of gastric cancer identifies subtypes associated with distinct clinical outcomes. *Nat. Med.* 21 449–456. 10.1038/nm.3850 25894828

[B12] Davila M. L. Riviere I. Wang X. Bartido S. Park J. Curran K. (2014). Efficacy and toxicity management of 19-28z CAR T cell therapy in B cell acute lymphoblastic leukemia. *Sci. Transl. Med.* 6:224ra25. 10.1126/scitranslmed.3008226 24553386 PMC4684949

[B13] Ding J. Wang K. Liu W. She Y. Sun Q. Shi J. (2016). Pore-forming activity and structural autoinhibition of the gasdermin family. *Nature* 535 111–116. 10.1038/nature18590 27281216

[B14] Ellis L. Z. Liu W. Luo Y. Okamoto M. Qu D. Dunn J. H. (2011). Green tea polyphenol epigallocatechin-3-gallate suppresses melanoma growth by inhibiting inflammasome and IL-1beta secretion. *Biochem. Biophys. Res. Commun.* 414 551–556. 10.1016/j.bbrc.2011.09.115 21982776 PMC3430966

[B15] Galon J. Bruni D. (2019). Approaches to treat immune hot, altered and cold tumours with combination immunotherapies. *Nat. Rev. Drug. Discov.* 18 197–218. 10.1038/s41573-018-0007-y 30610226

[B16] Gravalos C. Jimeno A. (2008). HER2 in gastric cancer: a new prognostic factor and a novel therapeutic target. *Ann. Oncol.* 19 1523–1529. 10.1093/annonc/mdn169 18441328

[B17] Hanzelmann S. Castelo R. Guinney J. (2013). GSVA: gene set variation analysis for microarray and RNA-seq data. *BMC Bioinformatics* 14:7. 10.1186/1471-2105-14-7 23323831 PMC3618321

[B18] He W. T. Wan H. Hu L. Chen P. Wang X. Huang Z. (2015). Gasdermin D is an executor of pyroptosis and required for interleukin-1beta secretion. *Cell Res.* 25 1285–1298. 10.1038/cr.2015.139 26611636 PMC4670995

[B19] Higgins M. J. Baselga J. (2011). Targeted therapies for breast cancer. *J. Clin. Invest.* 121 3797–3803. 10.1172/jci57152 21965336 PMC3195649

[B20] Hou J. Zhao R. Xia W. Chang C. W. You Y. Hsu J. M. (2020) PD-L1-mediated gasdermin C expression switches apoptosis to pyroptosis in cancer cells and facilitates tumour necrosis. *Nat. Cell. Biol.* 22 1264–1275. 10.1038/s41556-020-0575-z 32929201 PMC7653546

[B21] Hugo W. Zaretsky J. M. Sun L. Song C. Moreno B. H. Hu-Lieskovan S. (2016). Genomic and transcriptomic features of response to anti-PD-1 therapy in metastatic melanoma. *Cell* 165 35–44. 10.1016/j.cell.2016.02.065 26997480 PMC4808437

[B22] Ibrahim J. De Schutter E. Op de Beeck K. (2021). GSDME: a potential ally in cancer detection and treatment. *Trends Cancer* 7 392–394. 10.1016/j.trecan.2020.12.002 33422423

[B23] Jiang P. Gu S. Pan D. Fu J. Sahu A. Hu X. (2018). Signatures of T cell dysfunction and exclusion predict cancer immunotherapy response. *Nat. Med.* 24 1550–1558. 10.1038/s41591-018-0136-1 30127393 PMC6487502

[B24] Jiang Y. Zhang Q. Hu Y. Li T. Yu J. Zhao L. (2018). Immunoscore signature: a prognostic and predictive tool in gastric cancer. *Ann. Surg.* 267 504–513. 10.1097/sla.0000000000002116 28002059

[B25] John Hartigan M. W. (1979). Algorithm AS 136_ a K-means clustering algorithm. *Appl. Statist.* 28 100–108.

[B26] Li J. Byrne K. T. Yan F. Yamazoe T. Chen Z. Baslan T. (2018). Tumor cell-intrinsic factors underlie heterogeneity of immune cell infiltration and response to immunotherapy. *Immunity* 49 178–193e7. 10.1016/j.immuni.2018.06.006 29958801 PMC6707727

[B27] Li T. Kung H. J. Mack P. C. Gandara D. R. (2013). Genotyping and genomic profiling of non-small-cell lung cancer: implications for current and future therapies. *J. Clin. Oncol.* 31 1039–1049. 10.1200/jco.2012.45.3753 23401433 PMC3589700

[B28] Liu Y. Fang Y. Chen X. Wang Z. Liang X. Zhang T. (2020a). Gasdermin E-mediated target cell pyroptosis by CAR T cells triggers cytokine release syndrome. *Sci. Immunol.* 5:eaax7969. 10.1126/sciimmunol.aax7969 31953257

[B29] Liu Y. Wu J. Huang W. Weng S. Wang B. Chen Y. (2020b). Development and validation of a hypoxia-immune-based microenvironment gene signature for risk stratification in gastric cancer. *J. Transl. Med.* 18:201. 10.1186/s12967-020-02366-0 32410620 PMC7226948

[B30] Lossos I. S. Czerwinski D. K. Alizadeh A. A. Wechser M. A. Tibshirani R. Botstein D. (2004). Prediction of survival in diffuse large-B-cell lymphoma based on the expression of six genes. *N. Engl. J. Med.* 350 1828–1837. 10.1056/NEJMoa032520 15115829

[B31] Ma X. Guo P. Qiu Y. Mu K. Zhu L. Zhao W. (2016). Loss of AIM2 expression promotes hepatocarcinoma progression through activation of mTOR-S6K1 pathway. *Oncotarget* 7 36185–36197. 10.18632/oncotarget.9154 27167192 PMC5094992

[B32] Mariathasan S. Turley S. J. Nickles D. Castiglioni A. Yuen K. Wang Y. (2018). TGFbeta attenuates tumour response to PD-L1 blockade by contributing to exclusion of T cells. *Nature* 554 544–548. 10.1038/nature25501 29443960 PMC6028240

[B33] Mayakonda A. Lin D. C. Assenov Y. Plass C. Koeffler H. P. (2018). Maftools: efficient and comprehensive analysis of somatic variants in cancer. *Genome Res.* 28 1747–1756. 10.1101/gr.239244.118 30341162 PMC6211645

[B34] Newman A. M. Liu C. L. Green M. R. Gentles A. J. Feng W. Xu Y. (2015). Robust enumeration of cell subsets from tissue expression profiles. *Nat. Methods* 12 453–457. 10.1038/nmeth.3337 25822800 PMC4739640

[B35] Orning P. Weng D. Starheim K. Ratner D. Best Z. Lee B. (2018). Pathogen blockade of TAK1 triggers caspase-8-dependent cleavage of gasdermin D and cell death. *Science* 362 1064–1069. 10.1126/science.aau2818 30361383 PMC6522129

[B36] Park D. I. Yun J. W. Park J. H. Oh S. J. Kim H. J. Cho Y. K. (2006). HER-2/neu amplification is an independent prognostic factor in gastric cancer. *Dig. Dis. Sci.* 51 1371–1379. 10.1007/s10620-005-9057-1 16868827

[B37] Ritchie M. E. Phipson B. Wu D. Hu Y. Law C. W. Shi W. (2015). limma powers differential expression analyses for RNA-sequencing and microarray studies. *Nucleic Acids Res.* 43:e47. 10.1093/nar/gkv007 25605792 PMC4402510

[B38] Roepman P. Schlicker A. Tabernero J. Majewski I. Tian S. Moreno V. (2014). Colorectal cancer intrinsic subtypes predict chemotherapy benefit, deficient mismatch repair and epithelial-to-mesenchymal transition. *Int. J. Cancer* 134 552–562. 10.1002/ijc.28387 23852808 PMC4234005

[B39] Rogers C. Fernandes-Alnemri T. Mayes L. Alnemri D. Cingolani G. Alnemri E. S. (2017). Cleavage of DFNA5 by caspase-3 during apoptosis mediates progression to secondary necrotic/pyroptotic cell death. *Nat. Commun.* 8:14128. 10.1038/ncomms14128 28045099 PMC5216131

[B40] Ruschoff J. Dietel M. Baretton G. Arbogast S. Walch A. Monges G. (2010). HER2 diagnostics in gastric cancer-guideline validation and development of standardized immunohistochemical testing. *Virchows Arch.* 457 299–307. 10.1007/s00428-010-0952-2 20665045 PMC2933810

[B41] Shi J. Zhao Y. Wang K. Shi X. Wang Y. Huang H. (2015). Cleavage of GSDMD by inflammatory caspases determines pyroptotic cell death. *Nature* 526 660–665. 10.1038/nature15514 26375003

[B42] Shitara K. Ozguroglu M. Bang Y. J. Di Bartolomeo M. Mandala M. Ryu M. H. (2018). Pembrolizumab versus paclitaxel for previously treated, advanced gastric or gastro-oesophageal junction cancer (KEYNOTE-061): a randomised, open- label, controlled, phase 3 trial. *Lancet* 392 123–133. 10.1016/s0140-6736(18)31257-1 29880231

[B43] Smyth E. C. Nilsson M. Grabsch H. I. van Grieken N. C. Lordick F. (2020). Gastric cancer. *Lancet* 396 635–648. 10.1016/s0140-6736(20)31288-5 32861308

[B44] Sorlie T. Perou C. M. Tibshirani R. Aas T. Geisler S. Johnsen H. (2001). Gene expression patterns of breast carcinomas distinguish tumor subclasses with clinical implications. *Proc. Natl. Acad. Sci. U.S.A.* 98 10869–10874. 10.1073/pnas.191367098 11553815 PMC58566

[B45] Tanner M. Hollmen M. Junttila T. T. Kapanen A. I. Tommola S. Soini Y. (2005). Amplification of HER-2 in gastric carcinoma: association with Topoisomerase IIalpha gene amplification, intestinal type, poor prognosis and sensitivity to trastuzumab. *Ann. Oncol.* 16 273–278. 10.1093/annonc/mdi064 15668283

[B46] Thrift A. P. El-Serag H. B. (2020). Burden of gastric cancer. *Clin. Gastroenterol. Hepatol.* 18 534–542. 10.1016/j.cgh.2019.07.045 31362118 PMC8859863

[B47] Wang J. B. Li P. Liu X. L. Zheng Q. L. Ma Y. B. Zhao Y. J. (2020). An immune checkpoint score system for prognostic evaluation and adjuvant chemotherapy selection in gastric cancer. *Nat. Commun.* 11:6352. 10.1038/s41467-020-20260-7 33311518 PMC7732987

[B48] Wang Y. Gao W. Shi X. Ding J. Liu W. He H. (2017). Chemotherapy drugs induce pyroptosis through caspase-3 cleavage of a gasdermin. *Nature* 547 99–103. 10.1038/nature22393 28459430

[B49] Wilkerson M. D. Hayes D. N. (2010). Consensus clusterplus: a class discovery tool with confidence assessments and item tracking. *Bioinformatics* 26 1572–1573. 10.1093/bioinformatics/btq170 20427518 PMC2881355

[B50] Xia X. Wang X. Cheng Z. Qin W. Lei L. Jiang J. (2019). The role of pyroptosis in cancer: pro-cancer or pro-“host”? *Cell Death Dis.* 10:650. 10.1038/s41419-019-1883-8 31501419 PMC6733901

[B51] Yang Z. Liang X. Fu Y. Liu Y. Zheng L. Liu F. (2019). Identification of AUNIP as a candidate diagnostic and prognostic biomarker for oral squamous cell carcinoma. *EBioMedicine* 47 44–57. 10.1016/j.ebiom.2019.08.013 31409573 PMC6796785

[B52] Yeo J. G. Wasser M. Kumar P. Pan L. Poh S. L. Ally F. (2020). The extended polydimensional immunome characterization (EPIC) web-based reference and discovery tool for cytometry data. *Nat. Biotechnol.* 38 679–684. 10.1038/s41587-020-0532-1 32440006

[B53] Yoon H. H. Shi Q. Sukov W. R. Wiktor A. E. Khan M. Sattler C. A. (2012). Association of HER2/ErbB2 expression and gene amplification with pathologic features and prognosis in esophageal adenocarcinomas. *Clin. Cancer Res.* 18 546–554. 10.1158/1078-0432.Ccr-11-2272 22252257 PMC3261584

[B54] Yoshihara K. Shahmoradgoli M. Martinez E. Vegesna R. Kim H. Torres-Garcia W. (2013). Inferring tumour purity and stromal and immune cell admixture from expression data. *Nat. Commun.* 4:2612. 10.1038/ncomms3612 24113773 PMC3826632

[B55] Zaki M. H. Vogel P. Body-Malapel M. Lamkanfi M. Kanneganti T. D. (2010). IL-18 production downstream of the Nlrp3 inflammasome confers protection against colorectal tumor formation. *J. Immunol.* 185 4912–4920. 10.4049/jimmunol.1002046 20855874 PMC3104023

[B56] Zhang B. Wu Q. Li B. Wang D. Wang L. Zhou Y. L. (2020). m(6)a regulator-mediated methylation modification patterns and tumor microenvironment infiltration characterization in gastric cancer. *Mol. Cancer.* 19:53. 10.1186/s12943-020-01170-0 32164750 PMC7066851

[B57] Zhang Z. Zhang Y. Xia S. Kong Q. Li S. Liu X. (2020). Gasdermin E suppresses tumour growth by activating anti-tumour immunity. *Nature* 579 415–420. 10.1038/s41586-020-2071-9 32188940 PMC7123794

[B58] Zhou Z. He H. Wang K. Shi X. Wang Y. Su Y. (2020). Granzyme a from cytotoxic lymphocytes cleaves GSDMB to trigger pyroptosis in target cells. *Science* 368:eaaz7548. 10.1126/science.aaz7548 32299851

