# Supplementary material for: The Pyroptosis-Related Signature Predicts Prognosis and Indicates Immune Microenvironment Infiltration in Gastric Cancer
Source: Front Cell Dev Biol. 2021 Jun 11;9:676485. doi: 10.3389/fcell.2021.676485 (PMC8226259; doi:10.3389/fcell.2021.676485)
Supplement: Supplementary file 1 [file Data_Sheet_1.DOCX]

Supplementary Material

# Supplementary Tables

Table S1. Oligo sequences used in the real-time PCR

Table S2. GC classification pattern mediated by 11 pyroptosis-regulated genes in four GEO cohorts.

Table S3. GSVA enrichment analysis between these distinct pyroptosis-regulated clusters.

Table S4. DEGs between cluster 1 and cluster 2.

Table S5. Univariate analysis about DEGs.

Table S6. Multivariate analysis about DEGs.

Table S7. GSVA enrichment analysis between two different PS-score subgroups.

# Supplementary Figures


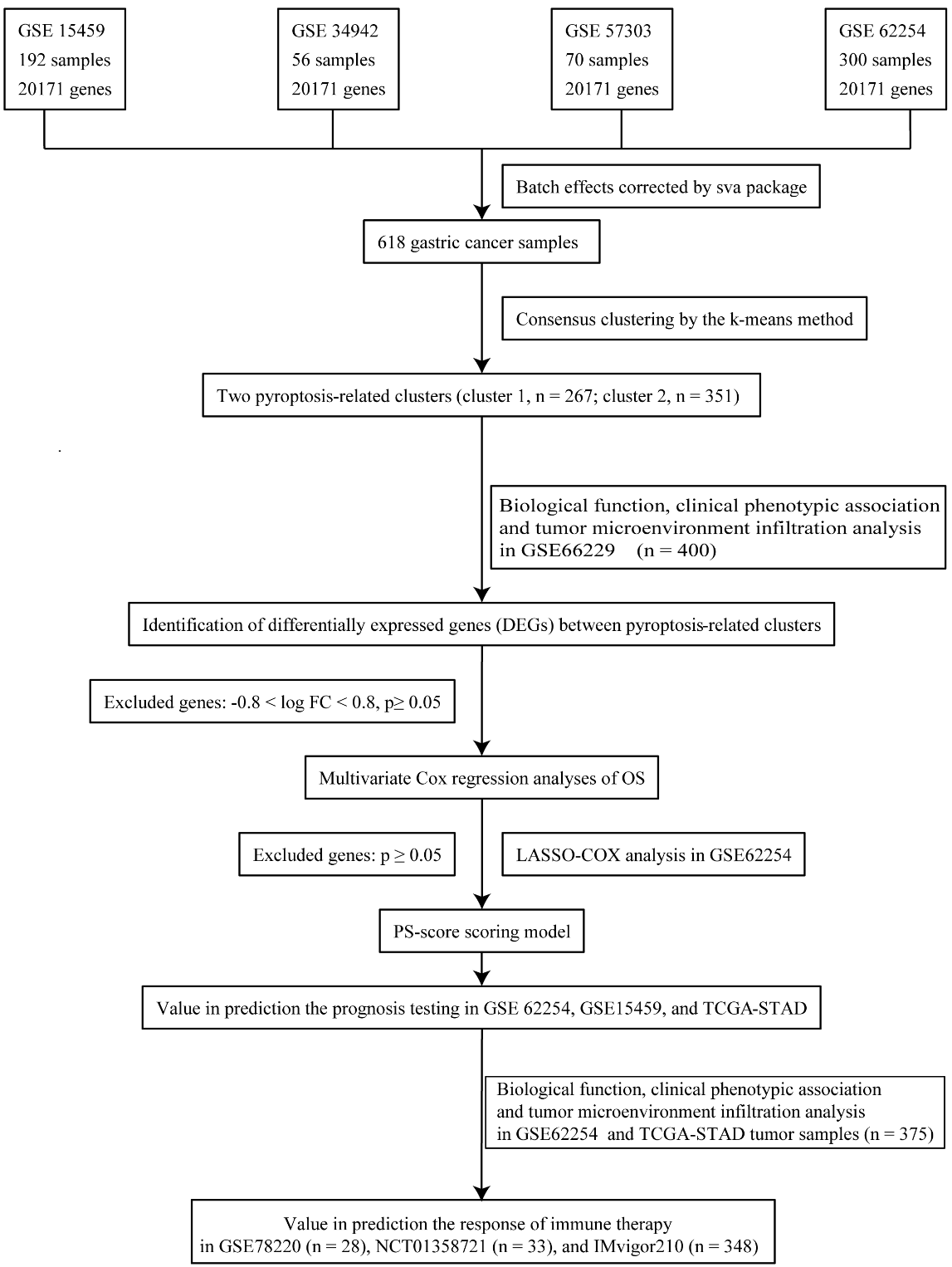


**Figure S1.** The workflow chart about each stage of statistical analysis. OS, overall survival


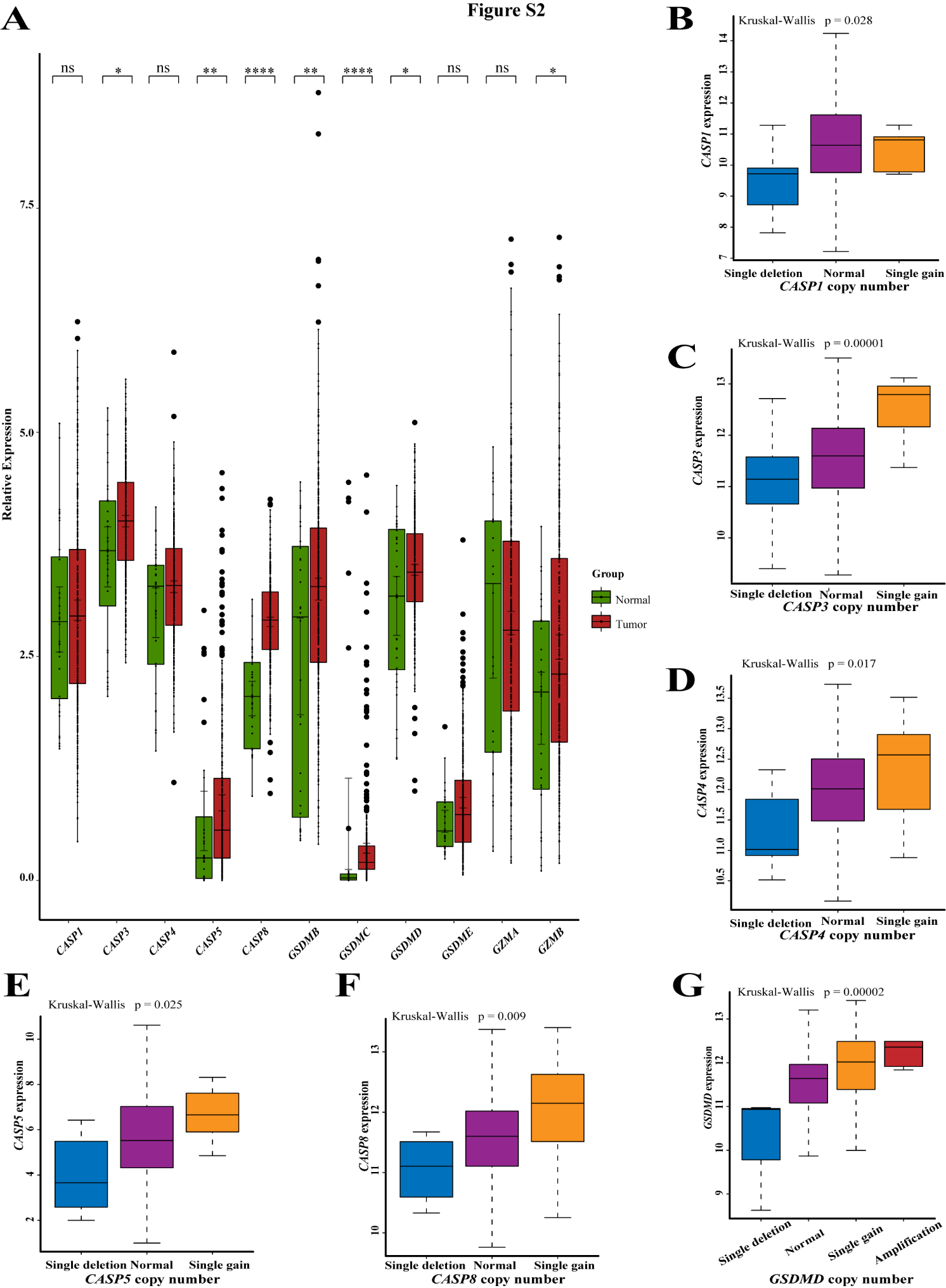


**Figure S2.** Expression of pyroptosis-related regulators with changes in copy number.

A. The expressions of pyroptosis-related regulators between normal samples (n = 32) and tumor samples (n = 375) of TCGA-STAD (Wilcox test, *, P < 0·05; **, P < 0·01; ****, P < 0·0001; ns, not statistically significant).

B-G. Changed copy numbers and their corresponding expressions of pyroptosis-related regulators in TCGA-STAD cohort (Kruskal-Wallis test, *, P < 0·05; **, P < 0·01; ****, P < 0·0001).


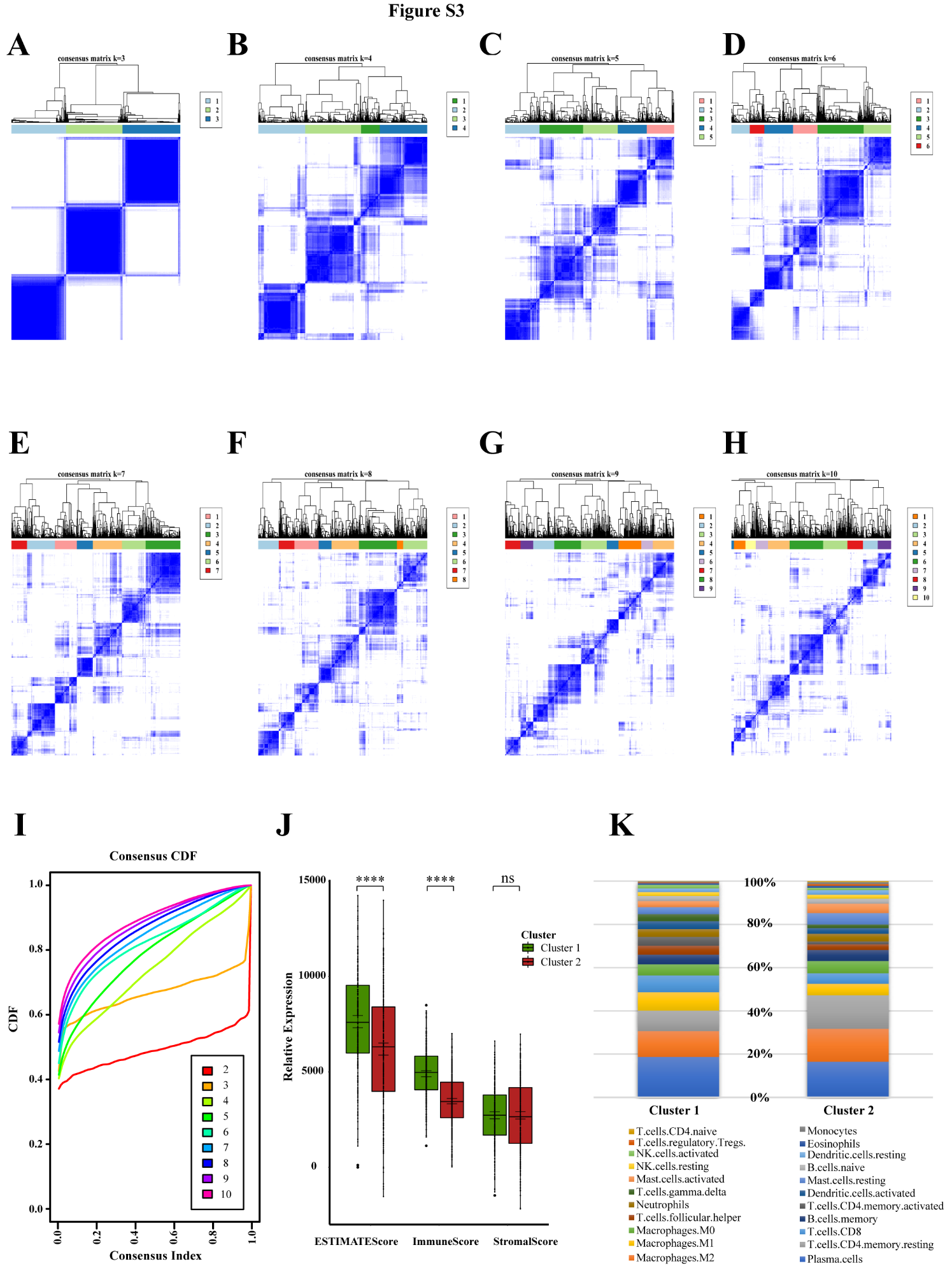


**Supplementary Figure 3.** Consensus clustering of pyroptosis-related regulators in gastric cancer by the k-means method.

A-H. Consensus clustering of 11 pyroptosis phenotype-related genes in GEO cohorts (GSE15459, GSE34942, GSE57303, and GSE62254) and consensus matrices for k = 3 - 10.

I. The consensus CDF curves were shown for different k from 2 to 10.

J. Different pyroptosis-related clusters indicated diverse immune scores by ESTIMATE in GEO cohorts (GSE15459, GSE34942, GSE57303, and GSE62254) (Wilcox test, ****, P < 0·0001; ns, not statistically significant).

K. The component differences of immune cells among the two pyroptosis-related patterns analyzed by CIBERSORTx in GEO cohorts (GSE15459, GSE34942, GSE57303, and GSE62254).


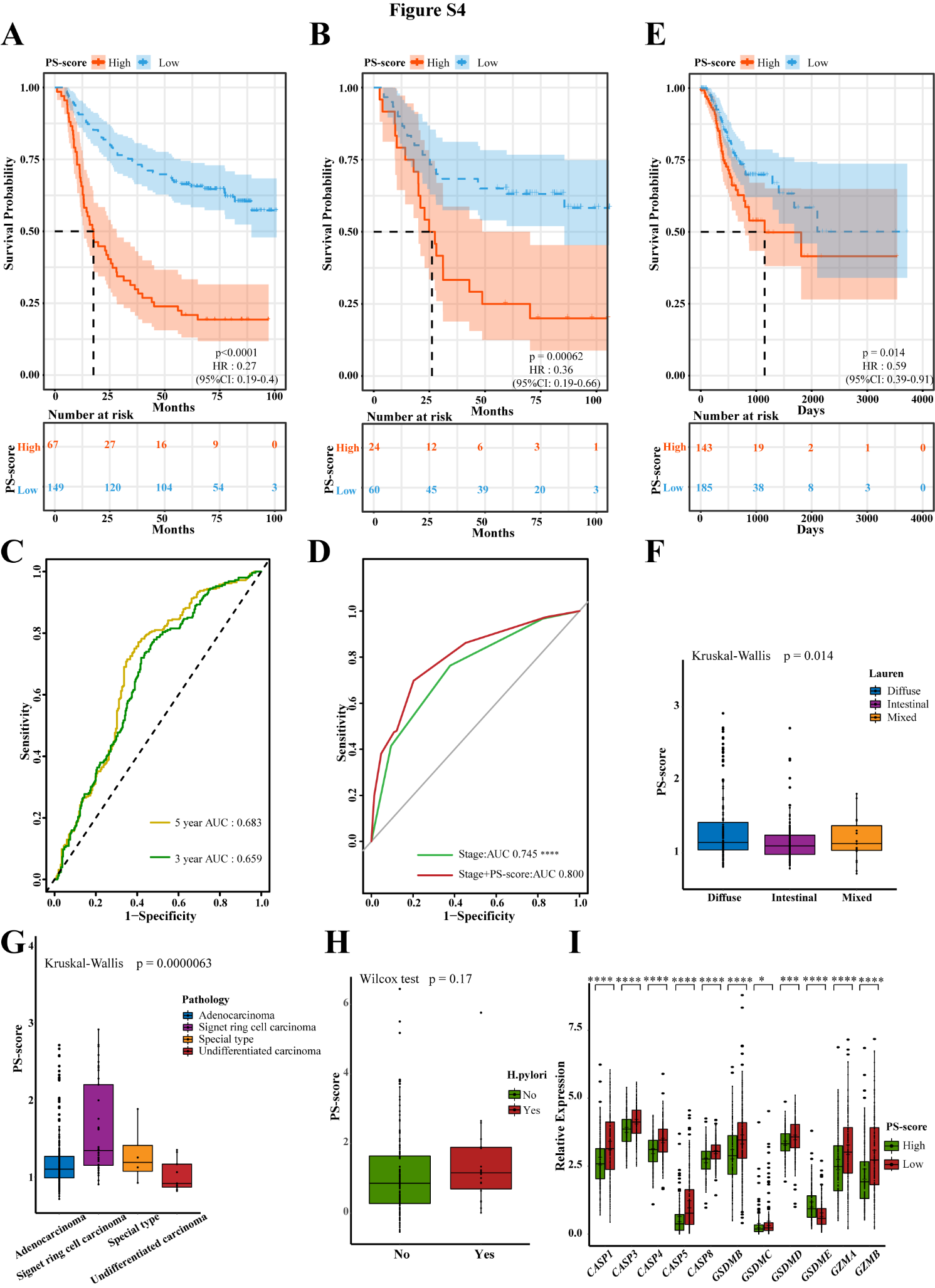


**Supplementary Figure 4.** Characteristics of PS-score subgroups.

A-B. OS curves for the training cohort (n = 216) and the testing cohort (n = 84) about PS-score with the cutoff value 1·258 in GSE62254 cohort (Log-rank test, the training cohort p<0·0001; the testing cohort p = 0·00062).

C. The time-dependent receiver operating characteristic (ROC) analysis of the PS-score. The area under the curve (AUC) was 0·659, 0·683 at 3 years, and 5 years, respectively in the above four GEO cohorts.

D. ROC curves about TNM stage with or without combining PS-score in GSE 62254 cohort with 0·745 and 0·800 AUC respectively (Mann Whitney tests; compared with Stage, ****, P < 0·0001).

E. DSS curves for the PS-score with the cutoff value 1·258 of gastric cancer samples in TCGA-STAD cohort. (Log-rank test, p = 0·014). DSS, disease-specific survival.

F-H. Various LAUREN types and pathological types revealed the difference in PS-score respectively in GSE62254 and *H.pylori* infection in TCGA-STAD (Kruskal-Wallis test, *, P < 0·05; ****, P < 0·0001; Wilcox test, *, P = 0·17).

I. Differential expression of pyroptosis regulated genes in low PS-score subgroup (n = 201) and high PS-score subgroup (n = 149) of TCGA-STAD cohort (Wilcox test, *, P < 0·05; ***, P < 0·001; ****, P < 0·0001).


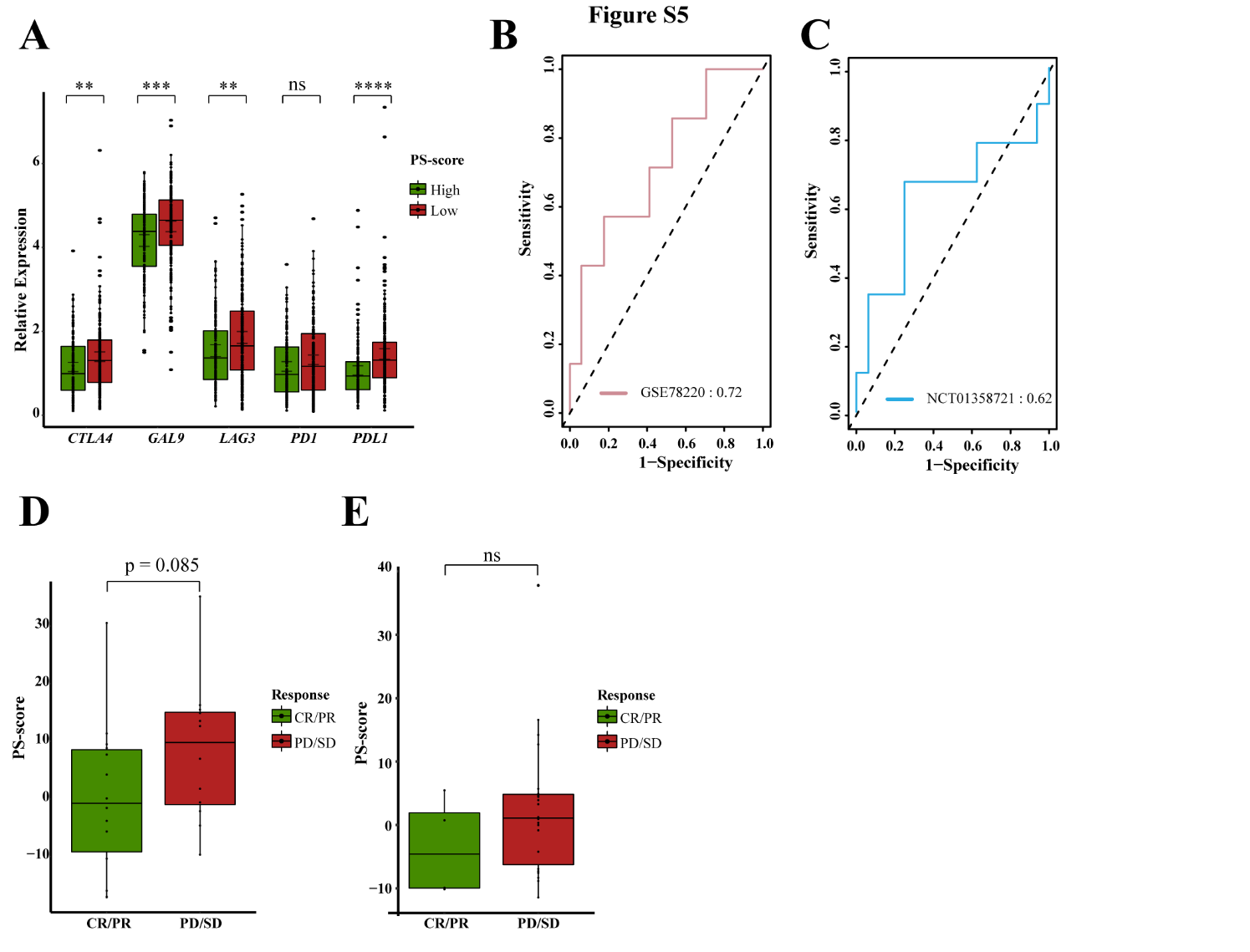


**Supplementary Figure 5.** Overview of PS-score in various clinical phenotypes and immune therapy responses.

A. Differential expression of immune checkpoint genes in low PS-score subgroup (n = 201) and high PS-score subgroup (n = 149) of TCGA-STAD cohort (Wilcox test, **, P < 0·01; ***, P < 0·001; ****, P < 0·0001; ns, not statistically significant).

B-C. ROC curves about PS-score in GSE78220 and NCT01358721 respectively.

D. Different PS-score in CR/PR group (n = 14) and PD/SD group (n = 12) in GSE78220 cohort (Wilcox test, P = 0·085). SD, stable disease; PD, progressive disease; CR, complete response; PR, partial response.

E. Different PS-score in CR/PR group (n = 8) and PD/SD group (n = 25) in NCT01358721 cohort (Wilcox test, ns, not statistically significant).
